# Inheritance of Refractive Error in Millennials

**DOI:** 10.1038/s41598-020-65130-w

**Published:** 2020-05-18

**Authors:** Dibyendu Pusti, Antonio Benito, Juan J. Madrid-Valero, Juan R. Ordoñana, Pablo Artal

**Affiliations:** 1 Laboratorio de Óptica, Instituto Universitario de Investigación en Óptica y Nanofísica, Universidad de Murcia, Campus de Espinardo (Ed. 34), 30100 Murcia, Spain; 2 Registro de Gemelos de Murcia, Departamento de Anatomía Humana y Psicobiología, Universidad de Murcia, Murcia, Spain; 3 Instituto Murciano de Investigación Biosanitaria (IMIB-Arrixaca), Universidad de Murcia, Murcia, Spain

**Keywords:** Behavioural genetics, Behavioural genetics, Refractive errors, Refractive errors, Risk factors

## Abstract

Over the last decades, the prevalence of myopia has suddenly increased, and at this rate, half of the world’s population will be myopic by the year 2050. Contemporary behavioural and lifestyle circumstances, along with emergent technology, are thought to be responsible for this increase. Twin studies mostly reported a high heritability of refractive error across ethnicities. However, heritability is a population statistic and could vary as a result of changing environmental conditions. We studied the variance of refractive error in millennials with 100 twin pairs of university students in southeast Spain. The study population presented a high prevalence of myopia (77%). Statistical analysis showed the variance of refractive error in this group of young twins was mainly driven by the shared environment and, to a lesser extent, by additive genetic factors. We found an increase in myopia prevalence accompanied by a decrease in heritability in this sample of millennials in contrast with results from a previous generation group from the same ethnic origin.

## Introduction

Millennials are defined as the generation born between 1982 to 2000^1^. There is a growing interest in studying millennials to investigate any health risks related to modern lifestyle. One crucial aspect affecting their quality of life is ocular refractive error. In particular, there is an increasing progression of myopia, which may transform this condition as the leading cause of visual impairment in the near future. Over the last decades, we are observing an increase in global myopia prevalence. If myopia keeps progressing at this rate, half of the world’s population will become myopic by the year 2050^2^. Studies conducted among different ethnic populations showed the prevalence of myopia is generally very high mainly in East Asian countries^2–8^. Nevertheless, the prevalence is increasing all over the globe which also includes United States^9,10^ and European countries^11^.

Approximately 1300 million people are suffering from some form of visual impairment globally. Eight out of ten of these visual disorders are avoidable with treatments extending from simple means of optical correction and cataract surgery to long-term medical therapies. Visual impairment associated with myopia can be due to uncorrected refractive error or underlying myopic maculopathy. Increased incidence of high myopia may lead to secondary ocular pathologies like myopic maculopathy, neovascularization, retinal detachment, cataract and increased risk of glaucoma^12–18^. These associated pathologies may lead to irreversible vision loss resulting in a higher prevalence of visual impairment. A recent study^2^ showed that the prevalence of myopia is highest among the 20 to 39 years age group worldwide, which is predicted to maintain a similar trend at this rate of progression. Some studies also linked the level of education with increased myopia progression^19,20^.

Heritability is static of how well differences in people’s genes account for differences in their phenotypic traits^21^. Twin studies are considered the most efficient approach to evaluate the heritability of a known phenotype^22^ and to model its genetic and environmental variance^23,24^. This model can estimate the variance of a known phenotypic trait, using similarities within monozygotic (MZ) or identical twin pairs compared to dizygotic (DZ) or non-identical twin pairs. Within a classical twin study, using Structural Equation Modelling (SEM), the variance of any phenotypic trait can be decomposed into four latent factors. They are: Additive (A) genetic variance represent the combined individual effects of alleles influencing a phenotype; Dominant (D) or non-additive genetic effects capture the variance due to interactions between genes, including dominance and, possibly, epistasis; Common (C) or shared environmental influences are those that are shared by the twins and act to make them similar to each other; unique or unshared Environment (E) impacts on each individual separately making twins in a pair different (it also includes measurement error). The division of each of these components by the total variance yields the different standardized components of variance, including heritability^25^.

Increased outdoor activity seems to be delaying the onset of myopia development during childhood^26,27^. Studies showed outdoor activities are not able to pull back myopic shift or may not be very effective in decreasing myopia progression in those who are already myopic^27–29^. However, the amount of time spent on outdoor activities may act as a factor as increased time spent outdoor in children showed slowing down the myopia progression^30,31^. Studies also showed that, regardless of these environmental influences, myopia still maintains a substantial genetic influence with higher odds ratio with parental myopia^32,33^. In this context, previous studies on heritability of refractive error showed a strong genetic impact^34–39^. The heritability for myopia has not been examined in a context of an intensified myopigenic environment. Hypothetically, the expanding of environmental conditions favouring myopia (regardless of genetic susceptibility) could reduce the relative impact of genetic factors on population variance.

To further understand this issue, we have performed a twin study on the heritability of refractive error among young university twins, who are at a high risk of myopia development. We included a sample of young students at the University of Murcia (Spain). Most of the previous twin studies have been performed among children, old or mixed twin population samples^35–38^. We aimed to analyze to what extent the variance of refractive error could be attributable to genetic or environmental factors in a sample of young university students.

## Results

**Figure 1:**
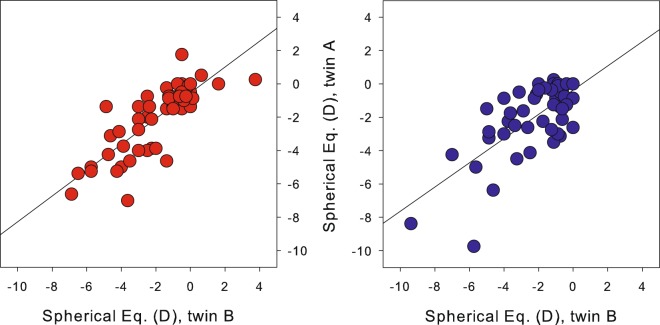
ICC of manifest refraction (SE; in D) for MZ couples (left; red) and DZ couples (right; blue).

A group of 200 university students were included in this study, corresponding to 54 monozygotic (MZ) or identical twin and 46 dizygotic (DZ) or non-identical twin pairs. Average spherical equivalent (SE) from manifest refraction was −2.0 ± 2.0 dioptres (D; range: +3.8 to −7.0 D) for the MZ group and −2.2 ± 2.1 D (range: 0.0 to −9.8 D) for the DZ group, with 77% of the subjects having myopia (see ‘Twin population’ in the Methods section for further detail). The intraclass correlation coefficient (ICC) between siblings for SE (Fig. 1) showed a similar correlation for both groups: 0.78 for MZ twins and 0.71 for DZ twins. Similar moderate-high correlations in both twin groups indicate a combination of genetic and shared-environmental impact on the selected trait.

**Figure 2:**
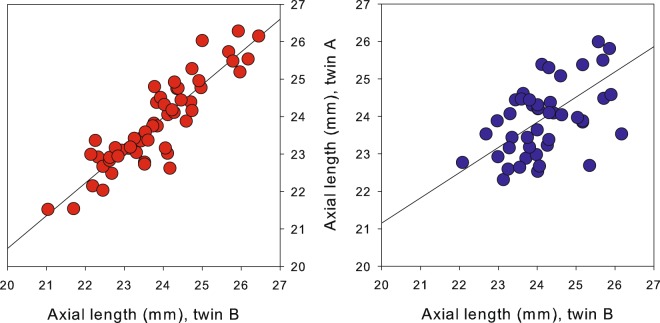
ICC of axial length (mm) for MZ couples (left; red) and DZ couples (right; blue).

Figure 2 presents the results for axial length (AL, in mm) for both MZ and DZ twin pairs. MZ twins showed higher ICC (0.88) than DZ (0.60) pairs. The higher ICC for AL in MZ twins, together with a moderate correlation in DZ, indicates the presence of genetic influence with additional shared-environmental influence supporting the SE results.

Given that correlations for DZ twins were always higher than half of correlations for the MZ twins, ACE models were fitted to the data for the estimation of variance components. Table 1 shows the results of the model fitting using Structural Equation Model (SEM), for the full ACE model as well as for more restricted, nested models to explain the variance of SE. Although, a priori, dropping the genetic component (CE model) did not produce a significant deterioration of model fit, given that the reduced sample size advice for the use of a more stringent criterion (p < 0.1) and that the Akaike’s Information Criterion (AIC) for the ACE model is the lowest one, we selected the full ACE as the best fitting model. Then, one-fourth of the variance was explained by the additive genetic component (A), nearly half of the variance was explained by the shared environment of the siblings (C), while the rest can be explained by the unshared environment (E) and measurement error.

**Table 1:** The proportion of the variance for refractive error (SE).

Model	A (95% CI)	C (95% CI)	E (95% CI)	df	−2LL	AIC	P
ACE	0.25 (0.00,0.61)	0.55 (0.20,0.78)	0.20 (0.13,0.32)	194	769.40	381.40	
AE	0.80 (0.71,0.87)	—	0.20 (0.13,0.29)	195	777.24	387.24	0.005
CE	—	0.74 (0.63,0.81)	0.26 (0.19,0.37)	195	772.23	382.23	0.09
E	—	—	1 (1,1)	196	850.32	458.32	<0.001

(A = additive genetic component; D = non-additive genetic component; C = shared environment; E = unique environment and error; df = degrees of freedom; −2LL = Twice the negative log-likelihood; AIC = Akaike’s Information Criterion).

## Discussion

**Figure 3:**
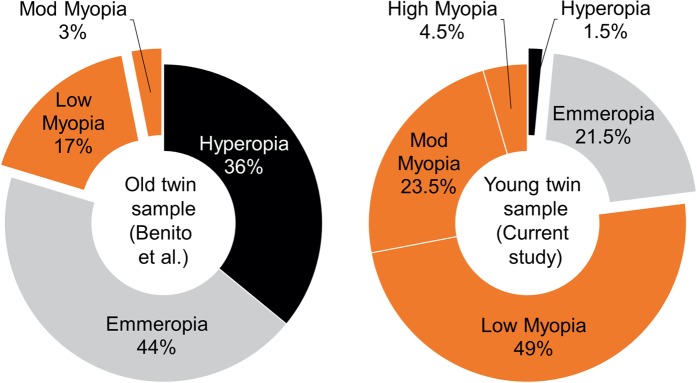
Change of refractive error distribution in four decades in southeast Spain: left, sample of old twins born in the late fifties of the 20^th^ century (from Benito et al.); right, sample of young twins born in the late nineties of the 20^th^ century (current study). Grey: hyperopia (>+0.5 D); white, emmetropia (SE lower than ±0.5 D); orange, myopia (low, −0.5 D to −3 D; moderate, −3 D to −6 D; high, <−6 D).

We found a high prevalence of low and moderate myopia in a sample of young twins (average year of birth was 1995.8 ± 3.0). A previous similar study was performed in a sample of twins born in the same geographical area, with an average age at the moment of the measurements (2014) of 54.9 ± 6.3 years (average year of birth was 1958.3 ± 6.7)^34,40^. On average, their manifest refraction gave a SE that was emmetropic (0.0 ± 1.4 D) for the MZ twins, and slightly hyperopic (+0.5 ± 1.6 D) for the DZ twins. This means a significant two-dioptre difference in average SE between the new young-twin sample and the previously measured older-twin one. Figure 3 represents the distribution of refractive error in the old twin and young twin samples used in the Benito *et al. (2016)* and the present study, respectively.

The highest discrepancy can be found in the prevalence of hyperopia, where young twins showed a fall by 24 times of old twins (1.5% from 36%). However, the higher prevalence of hyperopia in older subjects can be partially influenced by age-related decrease in the gradient index of the crystalline lens^41^. The percentage of emmetropia prevalence was dropped by 2 times (21.5% from 44% in young and old twins respectively).

On the other hand, the young sample showed 77% myopia prevalence with an increase of 3.9 times, while a much lower prevalence was observed in the old twin sample (20%). The present study sample showed increased myopia incidence by 7.8 times in case of moderate myopia (<−3.0 D to >−6.0 D) and by 2.9 times in low myopia (<−0.50 D to > −3.0 D). The present study sample showed 4.5% cases with high myopia (<−6 D), whereas the old twin sample showed none.

**Figure 4:**
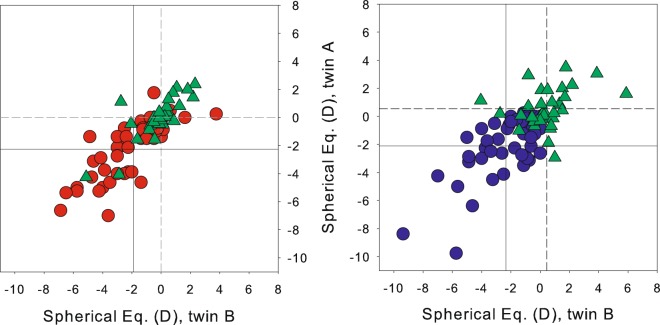
Change of manifest refraction ICC: same data as in Fig. 1, in addition to the results described in Benito *et al*.^34^ (green triangles) for MZ twins (left), and DZ twins (right). Solid lines represent mean SE in younger twins. Dashed lines represent mean SE in older twins.

Figure 4 shows the correlation of SE between the twin sample born in the middle of the 20th century and the present sample of young twins born in the late nineties (millennials). The older twin sample showed average emmetropic refraction with a higher correlation in MZ twin group than DZ (0.77 and 0.23, respectively). In contrast, the millennial twins showed a myopic shift (around two dioptres) with a close correlation for the MZ and DZ twin groups (0.78 and 0.71, respectively). The disparities found between twin samples could indicate that the variance of the manifest refraction can be influenced by different (kind or magnitude) environmental factors.

We found a change in the relative weight of factors explaining the variance of the manifest refraction: shifting from a heritability of 79% for the twins born in mid-20^th^ century to a heritability of 25% for those born in late-20^th^ century, what would indicate a change with an increasing role of environmental factors on the inter-individual variability. This is compatible with couples showing high refractive errors (outliers), as well as DZ couples in both young and old groups showing mixed refraction of one sibling being myopic and another hyperopic or emmetropic.

Table 2 summarises the heritability results of the twin studies conducted since 1962. It is important to note that not all these studies did use the same statistical approach to calculate heritability. Studies before 2001 mostly used a simpler method of heritability estimation using Falconer’s formula^22^; whereas, later on, SEM statistical analysis was used to explain phenotypic variances. Moreover, these studies were conducted across different timelines, age groups and ethnicities. The presented table is therefore to provide a comprehensive overview of results from previously published studies of refractive error inheritance. Table 2 also provides available data for myopia prevalence data in the cited studies.

**Table 2:** Summary of twin studies conducted since 1962.

Twin Study	No of twin pairs	Age	Myopia prevalence (%)	Heritability (%)
Sorsby *et al*.^53^ (1962)	MZ = 78, DZ = 40	4–14	NA	87
Kimura^54^ (1965)	MZ = 33, DZ = 16	15–20	NA	80
Nakajima^55^ (1968)	MZ = 39, DZ = 10	12–17	NA	83
Hu^56^ (1981)	MZ = 49, DZ = 37	7–19	NA	61
Lin and Chen^42^ (1987)	MZ = 90, DZ = 36	7–23	60	25
Teikari *et al*.^57^ (1991)	MZ = 54, DZ = 55	30–31	58	58
Angi *et al*.^43^ (1993)	MZ = 19, DZ = 20	3–7	35^#^	11
Lyhne *et al*.^39^ (2001)	MZ = 53, DZ = 61	20–45	25	90
Hammond *et al*.^38^ (2001)	MZ = 226, DZ = 280	49–79	26	84
Dirani *et al*.^36^ (2006)	MZ = 345, DZ = 267	18–88	25	82
Lopes MC *et al*.^35^ (2009)	MZ = 1152, DZ = 1149	16–82	28	77
Benito *et al*.^34^ (2016)*	MZ = 32, DZ = 32	46–72	20	79
The present study*	MZ = 54, DZ = 46	18–36	77	25

The twin study results are referred from a study by Guggenheim *et al*.^58^ with the addition of recently available data and the present study.

NA = dada not available

^#^Estimated from refractive error distribution histogram (SE) presented in the publication.

*Studies from the same ethnicity.

While most of the previous studies showed a large heritability for SE, some studies as those by Lin and Chen^42^ or Angi *et al*.^43^ reported low heritability in their tested twin samples. Focusing on those published in the last two decades, in 2001 Niels Lyhne *et al*.^39^ found 90% heritability for the age group from 20 to 45 years in Denmark. In the same year, Hammond *et al*.^38^ found 84% heritability in a sample of UK citizens with ages ranging from 49 to 79 years. Dirani *et al*.^36^ also found high heritability in a sample from Australia of 88% in men and 75% in women with ages ranging from 18 to 88 years. In 2009, Lopes *et al*.^35^ in another study conducted in the United Kingdom found 90% heritability in a twin group between 20 and 45 years. All these studies were conducted either across an old population or general population including all age groups except that by Lyhne *et al*.^39^ Furthermore, none of them captures the environmental changes that have occurred during the last two decades. The youngest subjects in the studied samples were born in the early nineties (Lopes *et al*., 2001) or mid-eighties (Dirani *et al*., 2006), and in both cases, their data is not analyzed separately but embedded within a larger sample including people of all ages. Consequently, our results are hardly comparable since ours is the only sample that has been raised during the early 21^st^ century.

The decrease in heritability and the increased myopia prevalence in our sample may not be related to the age of the sample, but with the year of birth and the changes in the environment in what these subjects developed during the last two decades. Due to the higher academic skills of our study population, it can be considered that they have been highly exposed to one of the myopia risks factors related to abuse of near work and large periods exposed to low luminance environments^11,19,20^. Many studies showed an increasing trend of myopia incidence as we are approaching the era of modern industrialization along with increasing education level^3,11,19,20^. The older twin sample^34^ had in average a lower education level and possibly less prolonged near task. This may signify the connection of these trends with our obtained results with greater environmental influence on refractive error variance among studied millennials. The change in myopia prevalence is happening across the last few decades, where the increased offset cannot be explained by a change on genetic factors, as genetic evolution cannot take place in such short time span. On the other hand, less than 10% of the variance of refraction could be accounted for Genome-wide studies, where studies have identified, so far, more than 150 single nucleotide polymorphisms (SNPs) associated with myopia^44^. Alternatively, the high heritability of refractive error observed in most of the twin studies can be a result of heritability overestimation by the classical twin study model. From any perspective, we must understand that heritability is the combined result of genotype and environment on a certain phenotypic trait. Hence, it appears that the sudden increase in myopia prevalence is a result of higher interaction with the environment. In contrast, the existence of feedback mechanism control over emmetropization has remained controversial. Angi *et al*.^43^ had measured child twin pairs (mean age was 5 years old) in Italy to evaluate the heritability of refractive defects during the ocular development stage. They found a very low heritability estimation of 8% to 14%, signifying the major influence of environment or visual feedback over the emmetropization process^37^. However they were most likely limited by the smaller sample size and lack of refractive error variation during ocular developmental stage in early years of life.

At the present study, we were limited with sample size which caused large confidence intervals. This power limitation also was a handicap for additional analyses comparing between groups. Hence, results should be interpreted with caution. Moreover, a questionnaire with detailed parental and personal history of our study subjects could have been an additional approach to gather extra information and analysis about their impact on refractive error, especially for the extreme cases.

In summary, we found a high prevalence of myopia among young university students together with a high environmental influence on the variance of refractive error (mainly myopia), which translates into a low heritability for the selected phenotypic trait: the spherical equivalent of the manifest refraction. Our result reflects a significant difference in comparison to previously published studies, in other words, between younger and older generations. The main difference in sample selection with most of the previous studies is possibly different education levels, modern lifestyle, and urbanization. We can explain our results as a possible environmental impact of increased near work in association with the modern era of industrialization and lifestyle.

## Methods

### Twin population

Twin pairs were recruited by the Murcia Twin Registry (Murcia, Spain) from the database of registered students of the University of Murcia during the study period from January 2017 to July 2018. Zygosity of the twins was confirmed by DNA analysis. Every subject was informed about the requirements and aims of the study, and a written informed consent obtained, following the tenets of the Declaration of Helsinki^45^. The ethics authorisation to perform the measurements was granted by the University of Murcia Research Ethics Committee. ID: 1108/2015. Exclusion criteria were: having an active ocular pathology or allergy, previous ocular surgery, ocular trauma, amblyopia or a decimal corrected distance visual acuity below 0.9. The right eye was considered in all cases as we found a very high correlation between both eyes. Table 3 shows the relevant data on the studied sample.

**Table 3:** Subject demographics and distribution of spherical equivalent of the manifest refraction.

	MZ (54 twin pairs)	DZ (46 twin pairs)
Age (yeas ± SD)	22.6 ± 4.0 (range: 19 to 30 years)	21.4 ± 2.4 (range: 19 to 36 years)
Mean foveal refraction (SE, D)	−2.2 ± 1.8 D (range: +3.8 to -7.0 D)	−2.2 ± 2.1 D (range: 0.0 to -9.8 D)
Emmetropia (%)	21	22
Myopia (%)	Total: 77	Total: 77
	Low: 46	Low: 52
	Mod: 27	Mod: 20
	High: 4	High: 5
Hyperpia (%)	2	1

Hyperopia: >+0.5 D; emmetropia: +0.5 D to −0.5 D; low myopia: −0.5 D to -3 D; moderate myopia: −3 D to −6 D; high myopia:>−6 D.

### Instruments

**Figure 5:**
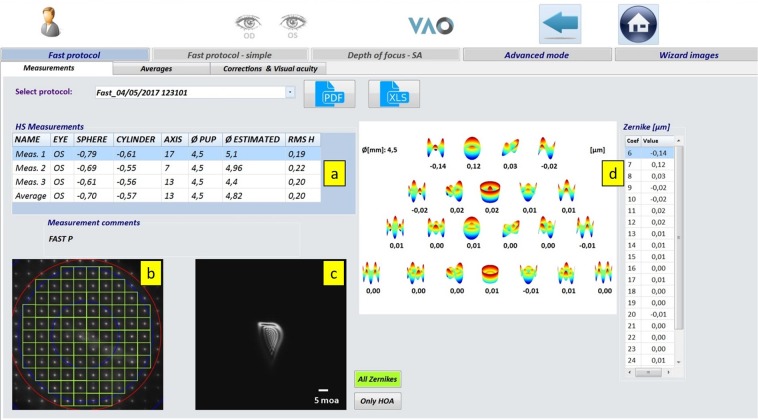
VAO objective refraction screenshot. (**a**) Results summary. (**b**) Raw HS image. (**c**) Simulated point spread function. (**d**) Higher-order Zernike coefficients.

We used the Adaptive Optics Visual Simulator (VAO; Voptica SL, Murcia, Spain)^46^ for visual acuity assessment and refractive error measurement under natural viewing conditions. VAO is an instrument that measures and corrects ocular aberrations using a liquid crystal programmable phase modulator. VAO can also perform routine clinical tests like visual acuity and monocular refraction. The instrument uses a near-infrared laser beam (780 nm diode laser) illuminates the eye and focuses on the retina. A Hartmann-Shack (HS) sensor measures the aberrations in real-time and controls the liquid crystal modulator in a closed loop. The aberration present in subjects’ eye is compensated or induced by activating the Liquid Crystal on Silicon (LCOS) spatial light modulator. The additional beam splitter placed behind the LCOS is used to allow the subjects to perceive a test with any desired modified aberration pattern. Test stimulus is displayed on a micro-display unit, presents wave-front guided optotypes or patterns to obtain subjective and objective results. The instrument can perform visual acuity test, objective and subjective monocular refraction, higher-order aberration (HOA) measurement, depth of focus curve by manipulating spherical aberration, simulation of optical profiles. However, in this study, we restricted our measurements to visual acuity and refraction measurements. Visual acuity and complete refraction process were performed for each twin subject and also used as a screening method to include study participants (*Fast assessment mode*, Fig. 5). Subjects were asked to look at a distance simulated target on the display unit of VAO during the objective measurements and used fogging technique to control accommodation during subjective refraction. Subjective or manifest refraction values were used in this study. The result summarizes measured refraction readings with average along with the raw HS image, the point spread function and higher-order aberration reading. Further detail about the instrument can be found ensewehere^47–49^.

Ocular biometry, including AL, was obtained by means of Lenstar LS900 (Haag-Streit AG, Köniz, Switzerland) ocular biometer. The instrument is designed to measure central (on-axis) axial length and IOL power calculation (biometry). In a single measurement scan and using optical low coherence reflectometry (OLCR) and can measure axial dimensions of all the ocular optical structures. Additionally, it measures keratometry or corneal curvature, pachymetry or corneal thickness, white-to-white or horizontal corneal diameter, lens thickness, anterior chamber depth, pupillometry or pupil diameter and more. In this study we have considered only axial length and average corneal radius of curvature values. The technical specification of the instrument along with further detail about this instrument can be found elsewhere^50^.

### Data analysis

Statistical analysis was performed using SPSS 24.0 (SPSS Inc. Chicago, IL) software. Normal distribution was checked by means of the Kolmogorov-Smirnov test. Differences between variables were obtained by means of the Student t-test for normally distributed and the U Mann-Whitney test for non-normally distributed variables. The intraclass correlation coefficient (ICC) was used instead of the Pearson correlation coefficient to avoid problems with twin data dependence while performing the comparison between siblings. To estimate the components of phenotypical variance (A, D, C and E) the data were analyzed using Structural Equation Modelling (SEM), using the Open Mx package in R^51^. C and D cannot be estimated at the same time in a classical twin study using only data from twins reared together. Hence the selection of a model including ACE or ADE components, is based on the pattern of twin correlations. An ADE model is usually selected when the DZ correlation is lower than half of the MZ correlation. In contrast, an ACE model is selected if the DZ correlation is greater than half of the MZ twin correlation^25^. Given the correlations pattern, only ACE models were estimated in this case. Mean effects of age and sex were added to the model as covariates to control their effect^52^.

## Data Availability

The datasets and codes used within this paper are available from the corresponding author upon reasonable request.
